# A Case of Acute Nephritis

**Published:** 1897-10

**Authors:** Ella M. Tuttle

**Affiliations:** New Berlin, N. Y.


### A CASE OF ACUTE NEPHRITIS.

Ella M. Tuttle, M. D., New Berlin, N. Y.

On the evening of October 7th, 1896, I was called in haste to a young woman who was said to be in labor and in need of my services at once. On reaching the house I found my patient (Mrs. A., aged twenty-four years, primapara) in bed, and very nervous and frightened, but having no labor pains. Examination revealed a tightly-closed cervix, though the woman was evidently at term. On questioning her I learned that she had suffered from sleeplessness and abdominal soreness all through the later months of her pregnancy. She had puffiness of the face and extremities, and some cloudiness of vision. I obtained a sample of the urine, gave her Ars. 3x, and left her.

On applying the usual tests to the urine, it was found to be loaded with albumen, becoming nearly solid on the application of heat. As may well be imagined, I felt very anxious during the few days that intervened before labor set in. Her sleep became better, and under *Puls.* 3x the abdominal soreness nearly disappeared, but the albumen persisted, and it was with great anxiety that I was summoned to her the morning of October 11th.

I found that labor had set in at 5 A. M. She was very nervous through it, and there were at times a twitching of the eyelids and drawing of the muscles of the mouth that made me fear a spasm was impending, but by giving the remedies that seemed indicated (chief among which was *Cimic.*) I delivered her at 2.30 P. M. of an eight-pound boy.

I thought that now the worst of the case was over and that the albumen would soon disappear, but it did not. On the contrary, it persisted, and under the miscroscope the urine showed numerous casts. The puffiness of the face and extremities increased, and the eyesight became so dim that she could with difficulty count fingers at a distance of three feet. There was a slight fever (101 degrees), persistent headache over the left eye, yellow-coated tongue, burning in the uretnra on urination. I now prescribed *Phos.* 4, with very gratifying results. In twenty-four hours the eyesight had begun to improve (though she complained of a red halo around the lamp), the headache had disappeared, and the albumen had sensibly diminished. From this time the case went on to an uneventful recovery, the only other remedies used being a few intercurrent doses of *Sulph.* 30, and a few doses of *Bell.* 3 for a threatened inflammation of the breasts. The cure seems to be permanent, for I examined the urine several months later, and found no signs of either albumen or casts.

---

The Editor was recently summoned to the case of a woman in a hospital who was safely delivered of a healthy child, but after a few hours went into convulsions. On our arrival we found her insensible and in the throes of death. Nevertheless we administered the indicated remedy, Opium. The patient was, however, too far gone to be rescued, and shortly afterward died. There was twenty per cent. albumen in the urine. Nothing was known of her previous history, as she had been admitted to the hospital but a few hours before the child was born.—ED.

